# Cardiac Wolframinopathies: A Case Report of Myocarditis and a Literature Review of Cardiac Involvement in Wolfram Syndrome 1

**DOI:** 10.3390/jcm13061803

**Published:** 2024-03-21

**Authors:** Andrea Villatore, Giulio Frontino, Maria Lucia Cascavilla, Davide Vignale, Davide Lazzeroni, Giovanni Peretto

**Affiliations:** 1 Disease Unit for Myocarditis & Arrhythmogenic Cardiomyopathies, IRCCS San Raffaele Scientific Institute, Vita-Salute San Raffaele University, 20132 Milan, Italy; 2 Division of Pediatrics, Diabetes Unit, Diabetes Research Institute, IRCCS San Raffaele Scientific Institute, Vita-Salute San Raffaele University, 20132 Milan, Italy; 3 Department of Ophthalmology, IRCCS San Raffaele Scientific Institute, Vita-Salute San Raffaele University, 20132 Milan, Italy; 4 Experimental Imaging Center, Radiology Unit, IRCCS San Raffaele Scientific Institute, Vita-Salute San Raffaele University, 20132 Milan, Italy; 5 IRCCS Fondazione Don Carlo Gnocchi—ONLUS, 50143 Florence, Italy

**Keywords:** diabetes, Wolfram syndrome, myocarditis, liraglutide

## Abstract

**Purpose**: Myocarditis is frequently a sporadic disease, but may also occur in the context of genetic disorders which may increase susceptibility to cardiac inflammation. Cardiac involvement in Wolfram syndrome type 1 (WS1) has been scarcely characterized. To our knowledge, no cases of virus-negative myocarditis have been reported in the WS1 pediatric population. **Methods**: We report the description of a pediatric case of acute myocarditis in the context of WS1, followed by a literature review of cardiovascular involvement associated with wolframin variants, and discuss potential pathophysiological mechanisms and therapeutic options. **Results**: A young patient with WS1, treated with insulin and liraglutide, was admitted for acute chest pain. Cardiac magnetic resonance and endomyocardial biopsy were performed to confirm the clinical suspicion of myocarditis. While congenital heart diseases and arrhythmias have been described previously in patients with WS1, this is the first description of virus-negative myocarditis. **Conclusions**: Myocarditis may represent a possible manifestation of cardiovascular involvement in WS1. Cardiovascular screening may be considered in patients with WS1.

## 1. Introduction

Acute myocarditis (AM) is an inflammatory cardiac disease, most frequently secondary to viral infections. However, pathogenic genetic variants in cardiomyopathic genes may account for a significant proportion of noninfectious AM overlapping with arrhythmogenic and dilated cardiomyopathy [1]. Advanced diagnostic techniques, multidisciplinary collaboration, and tailored follow-up may be necessary in this subgroup of patients to identify the disease’s etiology and define suitable treatment strategies. Wolfram syndrome 1 (WS1) is a rare monogenic, autosomal recessive disease, typically characterized by diabetes mellitus, diabetes insipidus, optic atrophy, sensorineural deafness, and other endocrine dysfunctions [2]. WS1 is an orphan disease with significant morbidity and mortality, often within early adulthood. However, some level of preclinical and clinical evidence of glucagon-like peptide-1 receptor agonists (GLP-1-RAs) efficacy in WS1 has been described in the literature [3]. While a number of cardiac abnormalities have been sporadically found in association with WS1, especially congenital heart defects, AM has never been reported to the best of our knowledge. The aim of our report is to describe a case of AM occurring in a young patient with WS1 and to present an updated review of the cardiac manifestations of the syndrome together with underlying pathophysiological mechanisms and possible therapeutic options.

## 2. Methods

We described a pediatric case of Wolfram syndrome 1 (WS1), presenting with acute myocarditis, who underwent gold-standard diagnostic work-up, including cardiac magnetic resonance and endomyocardial biopsy. Second, we performed a literature review of known cardiovascular involvement associated with wolframin pathogenic variants. Then, we discussed potential pathophysiological mechanisms. Finally, we elucidated the existing and forthcoming therapeutic strategies in WS1.

## 3. Results

**Figure 1:**
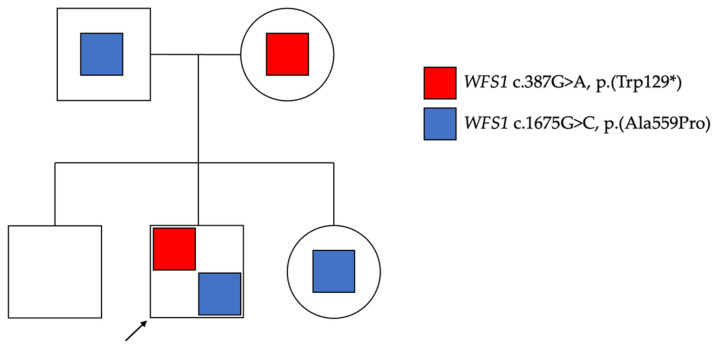
**Family pedigree.** The proband (arrow) showed a double heterozygous pathogenic variant in the *WFS1* gene: a nonsense variant, c.387G>A, determining a stop codon, p.(Trp129*), and a missense variant, c.1675G>C, p.(Ala559Pro). The variant c.387G>A was inherited from the mother and absent in the proband’s siblings, while the variant c.1675G>C was inherited from the father and also present in the proband’s sister.

A 16-year-old male, Caucasian, with an unremarkable family history, presented with WS1. Genetic testing confirmed a double heterozygous pathogenic variant of the *WFS1* gene: a nonsense variant, c.387G>A, determining a stop codon, p.(Trp129*), and a missense variant, c.1675G>C, determining an amino acid change, p.(Ala559Pro), which were consistent with autosomal recessive WS1. Family screening demonstrated that the variant c.387G>A was inherited from the mother and absent in the proband’s siblings, while the variant c.1675G>C was inherited from the father and also present in the proband’s sister. The family pedigree is shown in Figure 1.

**Figure 2:**
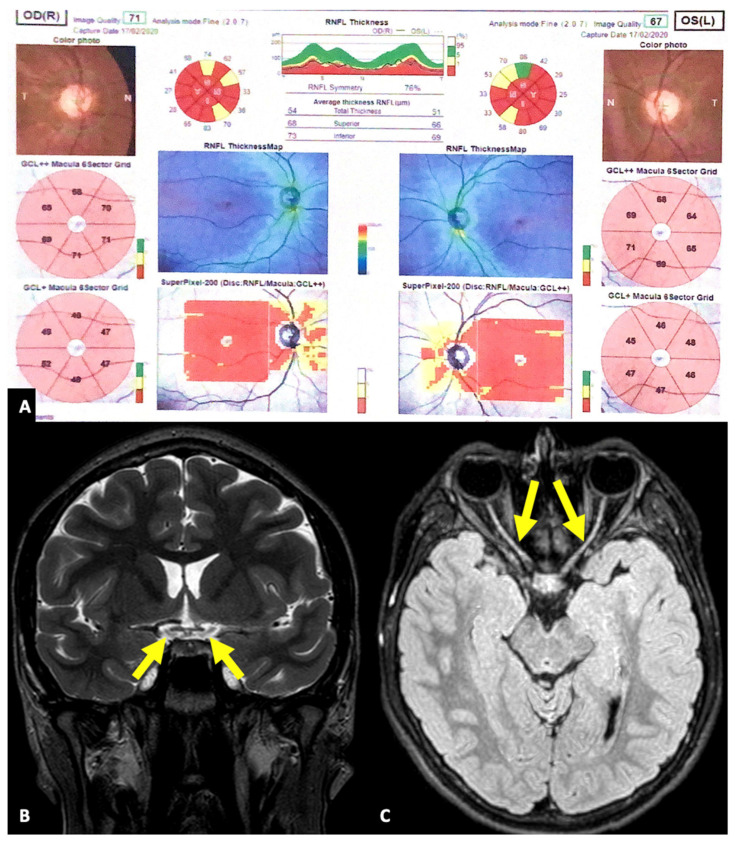
**Optic atrophy.** Optical coherence tomography (OCT) (**A**) showed a significant reduction in retinal nerve fiber layer (RNFL) thickness and extensive damage of ganglion cell layer (GCL). Brain magnetic resonance (MR), in the coronal T2 scan (**B**) and the axial fluid-attenuated inversion recovery (FLAIR) scan (**C**), showed marked thinning of the optic chiasm (arrows in (**B**)) and the optic nerves (arrows in (**C**)). Overall, OCT and brain MR were consistent with bilateral optic atrophy.

The patient initially had non-autoimmune type 1 diabetes mellitus (DM) and bilateral optic atrophy (see Figure 2). No other organ involvement was detected. The only ongoing therapy was that for DM, based on continuous subcutaneous rapid-acting insulin analog infusion with a hybrid closed loop system (Tandem Control-IQ and Dexcom G6), and off-label once-daily 1.8 mg liraglutide subcutaneous injection. At the last follow-up, glycemic control was optimal, with HbA1c 6.3% (45 mmol/mol).

Three years after WS1 diagnosis, the patient presented to the emergency department because of typical chest pain, fever (temperature 38 °C), and cough from the day before.

Objective clinical examination at arrival was unremarkable, with normal vital parameters. ECG showed sinus rhythm, normal atrioventricular conduction, ST-segment elevation from V3 to V6 without reciprocal changes, and a normal QTc interval. Troponin T peak was 659 ng/L (n.v. < 14 ng/L), NT-proBNP was 292 pg/mL (n.v. < 125 pg/mL), and C-reactive protein was 65.2 mg/L (n.v. < 6.0 mg/L). Blood glucose was in range. An extended panel for autoimmunity tested negative for underlying autoimmune diseases. In addition, viral serology did not show a systemic active infection. Finally, toxic causes, such as intoxications, venoms, and over-the-counter medicine, were excluded.

**Figure 3:**
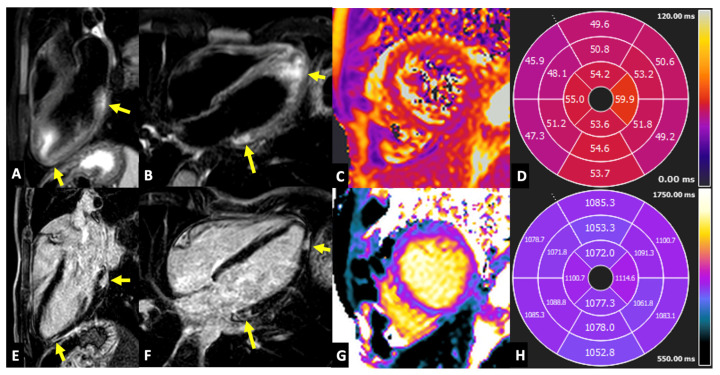
**Cardiac involvement.** Cardiac magnetic resonance, at the 3-chamber long axis (LA) (**A**) and 4-chamber LA (**B**). The STIR images show areas of hyperintensity, suggestive of focal edema, in the basal and apical inferolateral segments (arrows in (**A**,**B**)). T2 mapping (**C**) shows a segmental increase in T2 relaxation time (bull’s eye in (**D**); normal values < 50 ms), especially in the lateral and inferior walls from base to apex. The corresponding 3-chamber LA (**E**) and 4-chamber LA (**F**). The LGE images show the LGE with subepicardial distribution in the same areas of the edema (arrows in (**E**,**F**)). T1 mapping (**G**) shows a diffuse increase in T1 relaxation time (bull’s eye in (**H**); normal values < 1045 ms), which is more evident in the lateral wall.

The echocardiogram showed normal left ventricle (LV) and right ventricle (RV) volumes and systolic function, with a hyperechogenic left ventricle lateral wall, no significant valvular heart defects, and absent pericardial effusion. Cardiac magnetic resonance (CMR) confirmed normal biventricular volumes (LV end-diastolic volume 88 mL/mq; RV end-diastolic volume 89 mL/mq) and ejection fraction (EF) (LVEF 57%; RVEF 58%). Furthermore, CMR documented edema in T2-weighted short-tau inversion recovery (STIR) sequences and nonischemic late gadolinium enhancement (LGE), predominantly localized in the inferolateral wall, consistent with acute myocarditis (see Figure 3 for details). No signs of undiagnosed congenital heart disease were detected.

An endomyocardial biopsy (EMB) was performed to clarify the disease’s etiology and stage. Histology revealed structurally normal cardiomyocytes, absent necrosis, and rare interstitial lymphocytes (T CD3+ cells < 7/mm^2^), which did not fully meet the current diagnostic criteria for AM [4]. Unexpectedly, extensive replacement fibrosis was documented, suggesting a chronic underlying cardiomyopathic process. In keeping with a non-infectious preexisting disease, no genomes were detected via polymerase chain reaction (PCR) on myocardial tissue among the viruses that are commonly associated with myocarditis, including parvovirus B19 (PV-B19), adenovirus (AV), cytomegalovirus (CMV), Epstein–Barr virus (EBV), human herpes virus 6 (HHV6), herpes simplex virus (HSV), enterovirus/rhinovirus (EV/RV), and influenza virus.

Since chest pain resolved and no other complications were reported, namely systolic dysfunction on serial echocardiogram or arrhythmias on continuous ECG telemonitoring, the patient was discharged without any other therapy after 7 days of hospitalization.

At a 6-month follow-up, the patient was free from cardiac symptoms. Troponin T was normal. CMR was repeated, showing normal biventricular function, almost complete normalization of T2-weighted images, and a marked reduction in LGE. In the absence of relevant clinical endpoints, no immunomodulatory therapy was started. However, Holter ECG monitoring was repeated later on to exclude subclinical scar-related ventricular arrhythmias, and it documented no significant arrhythmic events.

## 4. Discussion

### 4.1. Wolfram Syndrome

WS1 is an autosomal disease due to pathogenic variants of the *WFS1* gene (chromosome 4p) encoding the protein wolframin, which is involved in membrane trafficking, secretion, processing, and regulation of endothelial reticulum (ER) calcium homeostasis [2,5]. WS1 is a very rare condition, with a prevalence ranging from 1 in 68,000 to 770,000 (4.8% to 0.57%) children. *WFS1* variants may have a dominant or recessive inheritance, including compound heterozygosity, and their clinical onset varies greatly in terms of both presentation and severity degree [6] Additionally, it is challenging to define genotype–phenotype correlations due to the molecular heterogeneity of WS1, the complex clinical features, and the limited dimension of patient populations [5].

Wolframin protein is highly expressed in brain tissues, pancreatic β-cells, and in the heart [7]. WS1 is principally characterized by early-onset non-autoimmune insulin-dependent diabetes mellitus, diabetes insipidus, optic atrophy, and sensorineural deafness [2,5]. Other neurological manifestations are cerebellar ataxia, dysarthria and dysphagia, psychiatric symptoms, and smell- and sleep-related abnormalities. Endocrine anomalies include anterior pituitary dysfunction, hypogonadism, and a deficit of growth hormone (GH). Gastrointestinal disorders account for bowel dysmotility, incontinence, and gastroparesis. Neurogenic bladder is a cause of hydroureteronephrosis, urinary incontinence, and recurrent infections. As conventional features, our patient had non-autoimmune insulin-dependent diabetes mellitus and optic atrophy but not diabetes insipidus [2,5].

### 4.2. Cardiovascular Involvement

In WS1 patients, heart diseases are considered very rare, but congenital heart diseases (CHD) and arrhythmias have been described [2,5]. Cases of heart diseases in WS1 from literature are reported in Table 1 The mechanism underlying the association between WS1 and cardiac malformations is still unknown. Several affected members of a WS1 family from Turkey showed ventricular septal defects (VSDs) [8]. Salzano et al. reported heart diseases in 14.3% of 14 patients, including VSDs, and secundum atrial septal defects (ASDs) with concomitant valvulopathy [9]. ASDs were also described in one of a pair of Chinese siblings [10]. In 16.1% of 31 Lebanese patients, valvular heart disease, particularly pulmonary stenosis, was described [11]. In 68 WS1 patients, only three cases had congenital cardiac anomalies, including Tetralogy of Fallot (ToF) in two and pulmonary valve stenosis in one. However, many patients also presented with sinus tachycardia and atrial and ventricular arrhythmias [12]. Aloi et al. described another ToF in a patient with compound heterozygous pathogenic variants of *WFS1* [13]. ToF was also described in 1 out of 24 patients from a Chinese cohort [14]. Another case of ToF was reported in a 5-year-old girl [15]. Heart disorders were mentioned as collateral manifestations in a study involving 23 Spanish WS1 patients [16]. Cyanotic CHD was described in another cohort from North India [17]. Finally, Wolfram-like syndrome due to the CDK13 variant segregating in a Pakistani family was associated with a bicuspid aortic valve and cardiac arrest in one case [18].

Multimodal diagnostic techniques, including CMR and EMB, and a multidisciplinary approach could be helpful for characterizing the features of “cardiac wolframinopathies”, that is to say, any abnormal cardiac condition associated with *WFS1* pathogenic variants, either congenital or acquired. Indeed, we show a case of concealed inflammatory cardiomyopathy in the setting of WS1. However, a definite causative pathophysiological link with wolframin loss of function cannot be assumed.

A viral infection may have been a possible trigger of myocarditis, as a second hit on genetic susceptibility. In fact, even though clinical presentation was preceded by flu-like manifestation, viral PCR on the myocardium tested negative. Since the clinical course was benign overall, we refrained from starting any cardiac therapy at the time, such as beta-blockers or anti-remodeling drugs for heart failure. Moreover, since inflammation spontaneously resolved at follow-up CMR, no targeted therapy for virus-negative myocarditis, namely immunomodulatory drugs, was started. However, in genetic forms, subsequent myocarditis relapses, evolution to dilated cardiomyopathy, and/or arrhythmias may be expected and will require a more attentive follow-up than patients with sporadic genotype-negative myocarditis.

### 4.3. Metabolic Alterations

Wolframin is a calmodulin (CaM)-binding protein, which regulates several calcium signal transduction processes through interaction with many cellular proteins. Therefore, WFS1, via control of the storage of cellular ER calcium, contributes to modulating cell apoptosis. Indeed, a 38-year-old woman with WS1 developed acute diffuse myocardial calcification during an episode of generalized sepsis due to calcium accumulation in cardiomyocytes. Remarkably, she had left ventricular dysfunction and signs of inflammation upon CMR [19].

Pathogenic variants of the *WFS1* gene could cause ER stress via alteration of cytosolic calcium homeostasis and subsequent impairment of mitochondrial dynamics in myocardial cells. The mitochondrial protein amount and activity were higher in Wfs1-deficient mice, as were mitochondrial proton leakage and oxygen consumption in murine skeletal muscle [20]. Further metabolomic studies in Wfs1-deficient mice revealed that perturbations in the metabolism of the heart occurred before the onset of related clinical signs, with the early intensification of glucose management but a later switch to lipolysis, as well as reduced cardiac glutathione content [21,22]. In cardiac muscle of Wfs1-knock-out (KO) rats, the primary change observed was the inhibitory effect of the activated fatty acid (FA) pathway on the utilization of the glucose-linked pathway. Wfs1-KO rats exhibit insulin deficiency without alterations in insulin sensitivity; therefore, the bioenergetic alterations in cardiac muscle could be secondary to either insulin deficiency or Wfs1 deficiency. The slight increase in cardiac muscle oxygen consumption observed could potentially be explained by a modestly elevated ratio of mitochondrial DNA to nuclear DNA, indicating increased mitochondrial content in the hearts of Wfs1-KO rats [23].

Other preclinical models were developed to recapitulate WS1, including a mouse [24] and later a rat that lacks the exon 5 of *Wfs1* (Wfs1^−e5/−e5^) and develops diabetes by nine months of age [25]. Consistent with the impact of Wfs1 deficiency independently of diabetic phenotype, the duration of cytosolic calcium transients as well as the duration and amplitude of contractile response were significantly prolonged in the left ventricular myocytes freshly isolated from the hearts of a four-month-old euglycemic animal [26]. These perturbations were not associated with the expression of SERCA2 on the protein level, but there was a significant reduction in both protein and mRNA expression levels of plasmalemmal sodium–calcium exchanger type 1 (NCX1) [27]. Hence, aside from its effects on ER stress, calcium levels, and mitochondria, wolframin also influences activities within the plasma membrane of myocytes.

### 4.4. Inflammation

Other CaM-related proteins, such as Ca^2+^/calmodulin-dependent protein kinase II (CaMKII), are already known to regulate inflammation in several cardiac diseases through nuclear factor kappa-B (NF-κB) signaling [28]. Another protein involved in calcium handling is phospholamban (*PLN* gene). Pathogenic variants in PLN are associated with arrhythmogenic and dilated cardiomyopathy, leading to ventricular arrhythmias and heart failure [29]. Therefore, CaM-related proteins, including WFS1, may represent another key target to modulate the entity of inflammatory-driven myocardial degeneration [29]. Remarkably, in our patient, extensive fibrosis was found at histology, associated with only rare lymphocytic infiltrates, suggesting subacute progressive myocardial damage.

Markers of inflammation and oxidative stress, such as IFN-γ, IL-1β, TNF-α, and isoprostane, were found to be elevated in patients with WS1 [30]. Another patient with recessive WS1 showed spontaneous high levels of pro-inflammatory cytokines produced by peripheral blood mononuclear cells (PBMCs) [31]. Silencing of the *WFS1* gene in healthy donors’ PBMCs led to a cytokine profile almost overlapping with WS1 patients. Furthermore, there was a dominance of pro-inflammatory Th17 over T regulatory (Treg) cells, as found in chronic inflammatory states [31]. Interestingly, a Treg/Th17 imbalance is also responsible for the development of myocarditis and dilated cardiomyopathy (DCM). [32] In detail, persistent heart failure in myocarditis was associated with high percentages of IL-17-producing T cells and IL-17-promoting cytokines, with significantly low percentages of FOXP3^+^ Treg cells, which may contribute to disease severity [33]. Inhibition of microRNA-155 ameliorated cardiac injury in an animal model of experimental autoimmune myocarditis via the suppression of Th17 immune response [34]. Therapies targeting interleukin pathways, such as anakinra, an anti-IL-1β, proved to be effective in myocarditis patients [1,35]. It is reasonable to hypothesize that immunomodulating drugs could also play a role in WS1 [31].

### 4.5. Therapeutic Opportunities

Currently, there are no specific treatments available to slow, stop, or reverse disease progression in WS1. Therefore, there is an ongoing endeavor to explore innovative therapeutic opportunities for WS1 [36].

Glucagon-like peptide-1 receptor agonists (GLP-1-RAs) are a promising treatment for WS1 patients. Interestingly, our patient was on off-label treatment with liraglutide, a GLP-1-RA, which proved to be safe and beneficial both in terms of residual C-peptide secretion and neuro-ophthalmological disease progression in a small series of WS1 patients [3]. The main mechanism seems to be the modulation of the dysregulated ER stress signaling that occurs in WS1 [3]. Other studies have demonstrated that exenatide and dulaglutide improved β-cell function by improving mitochondrial function, reducing oxidative stress, and preventing apoptosis in preclinical models of WS1 [37].

In addition, GLP-1RAs were shown to inhibit inflammatory responses and modulate the immune response, with specific activity on the Treg/Th17 axis [38]. Furthermore, preclinical evidence suggests that incretins suppress cardiac fibrosis and reduce inflammatory cytokine gene expression in a mouse model of experimental autoimmune myocarditis [39]. Even though controversial data about the safety of GLP-1-RAs in patients with advanced heart failure exist, our patient showed an uncomplicated course. Of note, despite liraglutide not being able to prevent myocarditis onset, we observed a prompt resolution of myocardial inflammation. Additional preclinical and clinical evidence is needed to investigate the potential immunomodulatory effects of liraglutide in inflammatory cardiomyopathies.

Sodium–glucose cotransporter 2 inhibitors (SGLT2i), as an adjunct to insulin, have also been proven to be effective and safe in type 1 DM [40]. Aside from the glucose-lowering properties, the cardioprotective effects of SGLT2i have been widely recognized among the entire spectrum of heart failure [41]. Remarkably, SGLT2i are able to reduce inflammation, oxidative stress, and fibrosis via several direct mechanisms, including decreases in pro-inflammatory cytokine production and fibroblast proliferation, the suppression of NLRP3 inflammasome and mTOR activation, the promotion of signaling through SIRT1, SIRT3, AMPK, and PGC1-alpha, the induction of autophagy and prevention of apoptosis, and the promotion of FAs and β-hydroxybutyrate [42]. To the best of our knowledge, SGLT2i have never been investigated in WS1, but could be an interesting option.

Another drug of interest is dantrolene sodium, which functions as an ER calcium stabilizer by targeting ryanodine receptor, an ER calcium transporter [43]. Moreover, treatment with dantrolene sodium has shown promise in reducing IL-1β and IL-21 levels after 6 months [36]. Valproic acid, by upregulating p21 expression and providing resistance against cell death, stimulates WFS1 expression and modulates the ER stress response in WS1 [44]. 4-phenylbutyric acid, a chemical protein folding and trafficking chaperone, restores normal insulin synthesis and insulin secretion regulation, by rescuing or stabilizing the original conformation of mutant WFS1 protein [45].

Regenerative therapy options utilizing induced pluripotent stem cells (iPSCs) have been investigated in order to replace abnormally functioning pancreatic β-cells and retinal ganglion cells [46]. Specific factors, such as mesencephalic astrocyte-derived neurotrophic factor (MANF), may stimulate β-cell proliferation and reduce ER stress [47].

Finally, gene therapy could represent a definite cure for WS1 patients. Utilizing adeno-associated viral (AAV) systems, wild-type *WFS1* could be introduced into defective organs to restore normal protein production. Moreover, CRISPR/CAS9 gene editing is another promising technology to correct the mutant *WFS1*.

## 5. Conclusions

To conclude, to our knowledge, this is the first report of myocarditis in the context of WS1. Cardiac involvement, accounting both for congenital heart disease and inflammatory cardiomyopathy, may present as a characteristic of WS1. Hence, cardiovascular screening may be considered in patients with WS1. Timely identification of cardiac conditions and proper intervention could mitigate the risk of cardiac-related complications.

## Figures and Tables

**Table 1:** **Cardiovascular (CV) involvement in WS1**. ASD = atrial septal defect. CHD = congenital heart disease. PVS = pulmonary valve stenosis. SVA = supraventricular arrhythmia. ToF = tetralogy of Fallot. VA = ventricular arrhythmia. VSD = ventricular septal defect.

Study	Number of Patients (% of Cohort)	CV (No. of Patients)	References
Bekir et al.	2 (20%)	VSD (2)	[8]
Salzano et al.	2 (14.3%)	VSD (1) and ASD (1)	[9]
Png et al.	1 (50%)	ASD (1)	[10]
Medlej et al.	6 (16.1%)	VSD (1) and PVS (5), plus cardiac autonomic dysfunction	[11]
Kinsley et al.	3 (4.4%)	ToF (2) and PVS (1), plus sinus tachycardia, SVA, and VA	[12]
Aloi et al.	1 (11.1%)	ToF (1)	[13]
Zhang et al.	1 (4.2%)	ToF (1)	[14]
Korkmaz et al.	1 (100%)	ToF (1)	[15]
Ganie et al.	1 (14%)	Cyanotic CHD (1)	[17]
Siani et al.	1 (100%)	Sepsis-induced myocardial calcification (1)	[19]
